# We have Vaccine for COVID-19! What to Recommend for Pregnant Women?

**DOI:** 10.1055/s-0041-1726090

**Published:** 2021-02-26

**Authors:** Silvana Maria Quintana

**Affiliations:** 1 Faculdade de Medicina de Ribeirão Preto, Universidade de São Paulo, Ribeirão Preto, SP, Brazil

On January 17, 2021, two vaccines were approved by ANVISA for emergency use to help combat COVID-19: Coronavac and Covishield/Oxford. The first vaccine is composed of inactivated (killed) viruses, was produced by the Chinese company Sinovac and in Brazil, will be produced by the Butantã Institute (São Paulo). The second is an adenovirus non-replicating viral vector vaccine and was produced by the pharmaceutical company Serum Institute of India in partnership with AstraZeneca/Oxford University. In Brazil, it will be produced by the Oswaldo Cruz Foundation (FIOCRUZ), Rio de Janeiro.

In view of the worrying and prolonged scenario of the COVID-19 pandemic, it is essential that Brazilian scientific societies share scientific knowledge free of political ideologies with their peers and position themselves based on the available evidence in relation to vaccines against COVID-19. In this context, gynecologists and obstetricians have requested guidelines on the indication of these vaccines in pregnant, puerperal and nursing women. I will highlight some points I consider important to support my opinion with which I will conclude this editorial.

Vaccines occupy a prominent position among the pillars of public health and allow both the eradication of diseases such as smallpox, and a significant reduction in diseases such as polio, rubella, tetanus and whooping cough, which used to be common in the past. 1 Two programs offered by the Brazilian Ministry of Health to the population through the National Health Service (Brazilian SUS) should be a reason for pride for all Brazilians: the STI/AIDS program and the National Immunization Program, active since 1973. The National Immunization Program of the Ministry of Health offers free of charge to the Brazilian population a set of vaccines of excellent quality that protect against 22 diseases, in addition to other immunobiologicals.

Vaccines are substances that aim to induce specific immunity by preventing invasion or eliminating pathogens circulating in the host or neutralizing microbial toxins, but without causing disease in the recipient. It is very important to know the composition of vaccines, especially to indicate their use in the pregnancy-puerperal cycle. Vaccines containing live (attenuated or modified) or inactivated (killed) antigen are considered safe techniques and have been known for more than 80 years. Recently, thanks to the evolution of scientific research, subunit or recombinant vaccines, vaccines using viral vectors, replicating or non-replicating, and nucleic acid vaccines such as messenger RNA (mRNA) have been developed.

Vaccines prepared with attenuated antigens contain the living but weakened form of the antigen, promoting a robust immune response with a prolonged duration, sometimes for the rest of life. The main representatives of this group are BCG, chickenpox, rubella, mumps, measles and yellow fever. As a general rule, they are not recommended during pregnancy. Inactivated vaccines consist of dead antigens after their exposure to chemical and physical agents and induce a less lasting immune response, requiring more doses to induce prolonged protection. Vaccines for influenza (flu), hepatitis A and rabies stand out in this group. All vaccines in this group can be used by pregnant women, but influenza vaccines are expressly recommended during pregnancy and the postpartum period. 2 Toxoid vaccines contain inactivated bacterial toxin and lead to weak immunization with need for a booster dose after a few years, such as tetanus and diphtheria vaccines, both recommended during the pregnancy-puerperal cycle. Recombinant vaccines are produced by genetic engineering techniques in which other agents are programmed to produce the desired antigenic fraction by stimulating a very effective immune response. Vaccines against hepatitis B and acellular pertussis are recombinant and recommended for application during pregnancy and the puerperal period. Viral vector vaccines insert a modified virus protein into another genetically weakened virus that is unable to replicate in the human body. When this vaccine is injected into the body, the immune system promotes an immune response to this protein that was hidden within the vector, leading to the production of antibodies and other defense cells capable of protecting the individual. Finally, nucleic acid vaccines such as messenger RNA (mRNA) contain a molecule with the SARS CoV-2 genetic code with instructions for cytoplasm ribosomes to initiate the synthesis of specific proteins of the surface of this virus. When these proteins are exposed to the immune system, they will be identified as an antigen, thereby triggering the immune response with the production of antibodies. There are still no controlled studies on the use of these vaccines in pregnant women. The National Immunization Program of the Ministry of Health recommends that pregnant women routinely receive three vaccines to protect against five diseases: Hepatitis B, DPT (diphtheria, pertussis, tetanus) and influenza. If needed, other vaccination schedules should be individualized and discussed with the pregnant/puerperal woman.

After the authorization for emergency use of vaccines for COVID-19 by ANVISA, at this time for priority groups such as health professionals, it is natural to question whether we should/can recommend these vaccines for women in the pregnancy-puerperal cycle and/or breastfeeding their children. Although the published clinical trials on vaccines against COVID-19 indicate safety and efficacy in the populations evaluated, there is no data on the immunogenicity, efficacy or safety of these vaccines in women in the pregnancy-puerperal cycle, as no clinical trial for these vaccines included this population. However, I emphasize that the Coronavac vaccine contains inactivated (killed) viruses, a technique with proven safety during the pregnancy-puerperal cycle for several years. In addition, the official package insert of this vaccine classifies the product as class B for use during pregnancy, that is, there are no studies in pregnant women, but studies in animals have not shown fetal damage. The other vaccine approved in Brazil, Covishield is a recombinant vaccine that uses chimpanzee adenovirus non-replicating viral vector technology. Although it is a relatively recent technique, in theory, the vaccine is safe for use during pregnancy.

Pregnant women experience important adaptations of their organism to provide adequate nutrition and oxygenation to the fetus, thus their predisposition to viral infections during this period. Studies comparing the behavior of COVID-19 in pregnant women with non-pregnant women showed that pregnant women who acquired SARS CoV-2 and developed symptoms of the infection were at a higher risk for: requiring hospitalization; developing severe conditions with admission to intensive care units (ICU); progressing to respiratory failure and requiring invasive ventilation (orotracheal intubation); and consequently, were at greater risk of death. In addition, from the obstetric point of view, higher rates of preterm birth and operative deliveries were observed. 3 4 5 In Brazil, data released by the Ministry of Health showed that SARS CoV-2 infection was the main cause of severe acute respiratory syndrome (SARS) in pregnant women in the third trimester and the mortality rate, especially in cases of concomitant comorbidities such as diabetes, hypertension and obesity, was alarmingly high. According to a study by FIOCRUZ and a group of Brazilian researchers, Brazil is the country with the highest number of deaths by COVID-19 in women in the pregnancy-puerperal cycle in the world. 6 7 Therefore, national and international data available so far allow to state that women during the pregnancy-puerperal cycle are part of the group at higher risk for complications from COVID-19, maternal death and unfavorable obstetric outcomes. In view of these results, in January 2021, the Ministry of Health released a technical standard 8 recommending the withdrawal of pregnant and postpartum women from face-to-face work given the important risks of COVID-19 to their health, reaffirming that they are a risk group for this infection.

Throughout these 11 months of the pandemic, the world population has been experiencing a major health crisis. We are facing a serious epidemiological situation with no prospect of a short-term resolution. When women in the pregnancy-puerperal cycle acquire SARS CoV-2 infection, they are at a higher risk for progressing to severe conditions and death, and this is even more relevant if they have comorbidities. The medications used to treat severe cases of this infection have not been tested for efficacy and safety in this group of women. In view of this scenario, the arrival of vaccines against COVID-19 brought hope of controlling the situation, but given the lack of information on efficacy and safety in pregnant and puerperal women, doubts arise regarding its recommendation. In this context, International Societies such as the American College of Obstetrics and Gynecology (ACOG), 9 the Society of Maternal-Fetal Medicine (SMFM) 10 and the Royal College 11 mentioned the need to include pregnant and lactating women in clinical trials, and recommended that vaccines against COVID-19 should not be denied to this group of women, especially if they are health professionals or have comorbidities. Note that the mRNA vaccine was the type released in these countries. In Brazil, the FEBRASGO Vaccine Commission 12 released a note that pregnant and lactating women belonging to the risk group may receive the vaccine after assessing the risks and benefits in a decision shared between the woman and her doctor.

Considering the increase in maternal morbidity and mortality associated with COVID-19 and previous experience with inactivated (killed) virus vaccines demonstrating the safety and efficacy of these immunobiologicals in pregnant women, the concern to indicate this vaccine due to the lack of data on efficacy and safety is not a justification for denying the vaccine to pregnant and puerperal women, especially those working as health professionals or who have comorbidities. In fact, this group should be considered a priority to receive vaccines against COVID-19.

Obviously, information about the lack of data on efficacy and safety, and about the performance of vaccines against COVID-19 in populations already studied should be clearly exposed to pregnant and puerperal women. The pregnant-puerperal woman's decision to be vaccinated must be taken after receiving this information and considering the risks of SARS-CoV-2 infection for pregnant women, their individual risk for COVID-19 infection and progression to serious illness (comorbidities, active health professional), and the vaccine safety. After these considerations, if the pregnant woman decides not to be vaccinated, her decision must be respected. The health professional must address the issue of vaccination in the prenatal consultation, including the vaccine against COVID-19 in addition to all vaccines recommended during pregnancy and the puerperal period, expose evidence-based information, assess the pregnant woman's risk and respect the woman's decision.

For puerperal and lactating women, who were also excluded from clinical trials, there are no data on the excretion of this substance in breast milk, but obviously, if puerperal women infected with SARS CoV-2 are allowed to breastfeed, that is, if the disease is compatible with breastfeeding, vaccination should be directed at these women. 13

Finally, it is a fact that pregnant women are at a higher risk of progressing to severe COVID-19 and that being a health professional or having comorbidities such as diabetes, obesity, hypertension can add risk to the lives of these women. In addition, a significant number of pregnant women will potentially be eligible to receive the vaccine against COVID-19 before the development of clinical trials to define efficacy and safety. We could lose many lives unless we adopt a more pragmatic position. I argue that pregnant women who have comorbidities or continue working as health professionals should be part of the priority group to receive the vaccine against COVID-19. The final decision whether or not to receive the vaccine will be made by the woman after receiving the appropriate information. This same principle applies with even greater emphasis to puerperal and lactating women. Regardless of the decision to vaccinate, antenatal care must be maintained and all pregnant women should receive guidance on infection prevention with emphasis on hand hygiene, social distance and wearing a mask.
